# Angiofibroma of Soft Tissue: A Newly Described Entity; A Case Report and Review of Literature

**DOI:** 10.7759/cureus.6225

**Published:** 2019-11-24

**Authors:** Zafar Ali, Fatima Anwar

**Affiliations:** 1 Histopathology, Shifa International Hospital, Islamabad, PAK

**Keywords:** angiofibroma, ema, soft tissue

## Abstract

Soft tissue angiofibroma is a relatively recent addition to the ever growing list of benign soft tissue tumors. It usually presents as soft tissue mass in the lower extremities in relation to joints and tendons. The tumor is composed of spindle-shaped fibroblastic cells with arborizing capillaries. We report a case of a 55-year-old female with a lump at the dorsum of left foot. Grossly the tumor was well circumscribed with yellow white cut surface. Microscopically the tumor showed typical features of angiofibroma with myxoid areas near the periphery of the lesion. Prominent vasculature is the integral part of the tumor with numerous small, branching, thin-walled blood vessels, accompanied by medium-sized ectatic vessels. Immunohistochemically the tumor cells are positive for epithelial membrane antigen (EMA). The location of the tumor, lack of cytological atypia, mitosis, and infiltrative margins help differentiate it from a sarcoma.

## Introduction

Angiofibroma of soft tissue is a recently described entity; it was first described in 2012 by Mariño-Enríquez and Fletcher as a benign fibrovascular tumor that resembles a low grade sarcoma [1]. These tumors typically present in the extremities as a slow growing painless lump. There is a slight female predilection. Grossly, these tumors are well circumscribed. The morphological features resemble those of angiofibroma of nasal cavity. On immunohistochemistry (IHC), the neoplastic cells are focally positive for epi­thelial membrane antigen (EMA); occasional cases show scattered cells that stain CD34, smooth muscle actin (SMA), and desmin. We present a case of angiofibroma at the dorsum of foot.

## Case presentation

**Figure 1:**
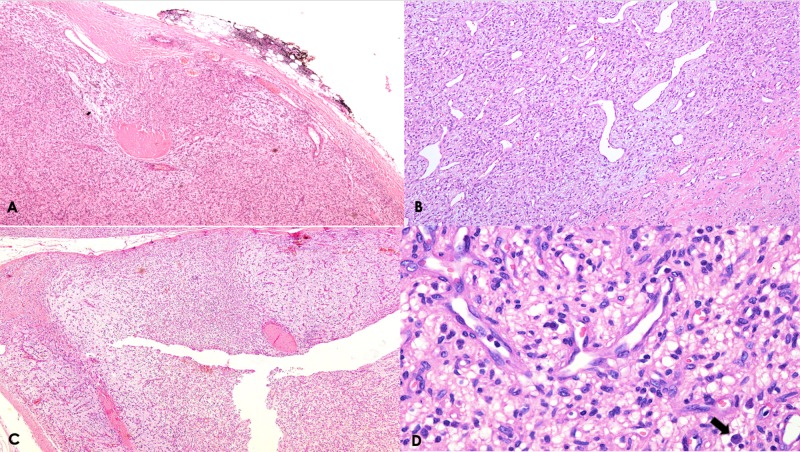
A: Low power view showing a well-circumscribed nodular lesion with relatively thick capsule (H/E, 40x). B: Prominent vascular pattern consisting of numerous small thin-walled vessels and occasional ectatic vessels (H/E, 200x). C: There is regional variation in cellularity with the stroma variably myxoid to collagenous (H/E, 40x). D: High power view showing uniform bland spindle cells with inconspicuous cytoplasm and ovoid or tapering nuclei. Occasional mast cells are seen, black arrow (H/E, 400x).

A 55-year-old female with no significant past history, presented with a slow growing, painless lump on the dorsum of left foot for the past few months. She underwent surgical excision at a private hospital and the specimen was sent to our histopathology department. On gross examination, it was found to be an unoriented nodule, weighing 13.8 g and measuring 3.5 cm x 3.2 cm x 1.5 cm. Cut surface was mucoid, tan yellow, soft to firm in consistency. Microscopically, it showed a circumscribed tumor showing proliferation of uniform bland spindled cells with inconspicuous cytoplasm and ovoid nuclei. Background was variably myxoid to collagenous with a prominent network of small thin walled and finely branched blood vessels. The tumor was reaching close to circumferential painted margins (distance less than and equal to 1.0 mm). There is regional variation in cellularity with the stroma variably myxoid to collagenous. The spindle cells are uniform bland with ovoid nuclei and inconspicuous cytoplasm. Stromal chronic inflammatory infiltrate and mast cells common (Figure 1A-D).

**Figure 2:**
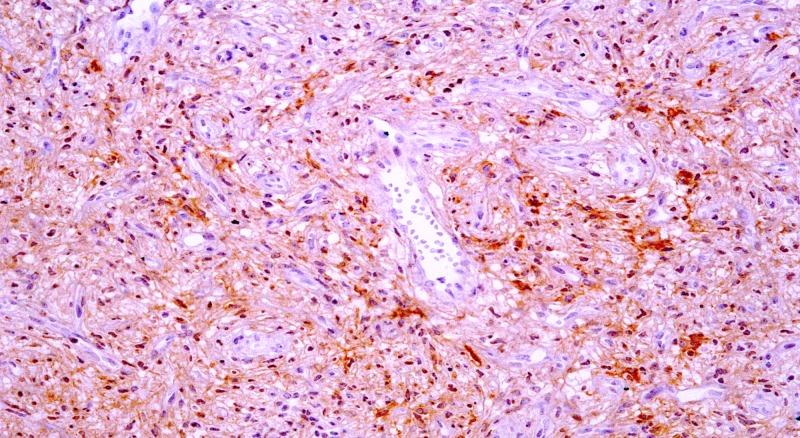
CD34 immunostain is positive in the tumor cells with variable staining intensity (200x).

On IHC, there was multifocal positivity for EMA, as quite often seen in these lesions, while the tumor cells were negative for SMA and S-100 protein (Figure 2).

A diagnosis favoring angiofibroma of soft tissue was made. The case was also sent to Dr. Fletcher for review and he also agreed with our interpretation.

## Discussion

Angiofibroma of soft tissue is a benign fibro­vascular neoplasm that was first described by Mariño-Enríquez and Fletcher in a series of 37 cases in 2012. It presents as slow growing, deep seated masses in the extremities, predominantly involving lower extremity. However, it can also occur in back, chest wall, abdominal cavity, and pelvic cavity [2]. There is female predominance affect­ing females twice as frequently as males according to the cases reported up till now [3]. Most of the lesions described by Fletcher et al. are well circumscribed having a median size of 3.5 cm. The lesion in our case measured 3 cm in maximum dimension.

Microscopically, the lesion is characterized by two components: a relatively uniform proliferation of bland, spindle-shaped cells with inconspicuous cytoplasm and ovoid-to-tapering nuclei set in a variably collagenous or myxoid stroma. A prominent vascular network composed of numerous small, branching, thin-walled blood vessels, often accompanied by medium-sized round or irregular and ectatic vessels can be seen. Occasional mitoses (1-4/10 HPF) may be present [4]. Mild degenerative nuclear atypia can be seen, however, it is uncommon. Our case showed a circumscribed tumor with proliferation of uniform bland spindled cells with inconspicuous cytoplasm and ovoid nuclei. Stroma was collagenous to myxoid and thin walled, finely branched blood vessels were seen. The tumor was reaching close to circumferential painted margin (distance less than and equal to 1.0 mm). Mitoses were infrequent in our case and no atypia was identified. Immunohistochemically, there may be focal expression of EMA, CD34, SMA, and desmin [5]. Our case also showed focal positivity for EMA while SMA and S100 were negative. Few cases, analyzed cytogenetically, have shown simple karyotype with a balanced t(5;8)(p15;q13) chromosomal translocation [6].

The differential diagnosis of angiofibroma of soft tissue includes vascular, spindle cell neoplasms such as solitary fibrous tumor (SFT), low-grade fibromyxoid sarcoma (LGFMS), and myxoid liposarcoma [7]. SFT can occur outside the pleu­ra, similar to that of angiofibroma, the spindle cells in SFT are cytologically bland arranged in pattern less growth architec­ture. In addition, regional variation in cellularity, thick collagenous fiber deposits, foci of myxoid degeneration, and large-sized branch­ing hemangiopericytoma-like vessels can also be noted in both the two tumors. However, SFT lacks the innumerable, evenly distributed, arbo­rizing thin-walled vessels which are characteristic of angiofibroma. Furthermore, the tumor cells in SFT typically show strong and diffuse expres­sion of CD34, whereas CD34 expression is variable in angiofibroma. STAT-6 is strongly positive in SFT while negative in angiofibroma. LGFMS typically affects young adults and presents as a larger and more deep­ly localized mass; it shares a bland spindle cell composition and alternating collagenous and myxoid areas with soft tissue angiofibroma. LGFMS shows t(7; 16) FUS-CREB3L2 or t(11;16) FUS-CREB3L1, unlike angiofibroma of soft tis­sue. In myxoid liposarcoma, bland spindle cell proliferation is seen with prominent plexi­form thin-walled capillaries, hence the pos­sible diagnostic confusion with angiofibroma of soft tissue. However, it shows scattered univac­uolar and multivacuolar lipoblasts throughout the lesion as well as stromal mucin pools which are not seen in angiofibroma of soft tissue.

Treatment includes simple or wide excision. The lesion may recur if excised incompletely [8]. Our patient underwent surgical excision and currently there is no evidence of recurrence.

## Conclusions

Angiofibroma which is a benign soft tissue tumor that may recur locally is incompletely excised. Knowledge of this entity is important as it can mimic other benign and malignant soft tissue tumors. Multifocal positivity of CD34 is quite often seen in these lesions and should not be confused with other CD34 positive tumors of skin and soft tissue.
